# Performance of single skin masonry walls subjected to hydraulic loading

**DOI:** 10.1617/s11527-018-1222-z

**Published:** 2018-07-11

**Authors:** D. M. Herbert, D. R. Gardner, M. Harbottle, T. G. Hughes

**Affiliations:** Cardiff School of Engineering, Cardiff University, Queens Buildings, Cardiff, CF24 3AA UK

**Keywords:** Masonry, Small scale, Hydraulic loading, Modelling

## Abstract

Property owners are facing increasing threats from flooding and in response are likely to turn to products designed to waterproof or ‘seal’ the outside of the building in an effort to prevent the ingress of flood water. However, very limited research has been conducted on the effect of this sealing action and the consequent hydraulic load acting upon the structure of the building. The theoretical safe application of waterproofing products has been suggested to be between 0.6 and 1 m (published guidance suggests 0.9 m), although the experimental evidence supporting these suggestions is either absent or limited in nature. This paper presents the findings of an experimental programme that has examined the effect of out-of-plane hydrostatic loading on masonry walls typical of domestic or commercial buildings. The study, conducted at 1/6th scale using a geotechnical centrifuge considers wall panels constructed from a variety of masonry units (autoclaved aerated concrete block, brick and brick-block) bound together with two different types of mortar. The wall panels were subject to an axial load representative of 1 storey of loading and were simply supported on all 4 sides. The load—out-of-plane deflection response of the panels was captured by a 3D digital image correlation system, and the water level at failure was compared to that predicted from previous research and the established yield line analysis method with encouraging results. When partial material and load factors were taken into consideration the results illustrated that a safe sealing height of 0.9 m, as quoted in the literature, would generally be inappropriate, whilst the safe sealing height of 0.6 m was not suitable for every case investigated. This supports the need for a suitable approach for the calculation of water levels at failure rather than the use of fixed values given in published literature.

## Introduction

Flooding damage to properties and their contents has generated significant attention in recent years, following extreme weather events in the UK and beyond. Approximately 2.75 million properties are at risk of flooding in England, Scotland and Wales [1–3] and increasing numbers of flood events are expected [4–7]. Together with reduced spend on flood defences [8] this contributes to estimated annual flood and storm damage costs of approximately £1.3bn [9], possibly rising to £27bn by 2080 [10]. With or without large flood protection schemes, property owners may therefore consider turning to products that will provide resilience or resistance to flood waters. Recommended changes in building regulations and government policy may encourage more common use of these systems [5].

Two major methods of avoiding flood damage are to use resilient materials and design, allowing the property to be quickly returned to a habitable state after flooding, or external flood resistant systems preventing floodwater entry. The latter systems (e.g. air brick seals, flood door barriers) frequently rely on the structure of the building to retain the water, but very limited work has been conducted to assess the implications of this method of flood protection. Experimental evaluation of the load capacity of a small number of masonry wall panels subject to non-uniform hydraulic lateral loads found that prototype-scale brick and block walls with the top edge unsupported failed at water levels of 0.73 and 1.07 m respectively [11]. Inclusion of an opening within the brick wall was found to have no significant effect on the failure load. A further test conducted of a brick wall with restraint at the top edge sustained a higher water level of 1.45 m, but was found to fail suddenly. Tests on a full-scale structure externally waterproofed with reinforced plastic sheeting found that at a water level of 0.91 m the structure could safely support the load, however at a level of 1.22 m permanent deformations and cracking were observed [12]. It was recommended that masonry buildings should be waterproofed to a maximum height of 0.91 m, although construction techniques used were relevant to North America, only one size of wall panel was considered and limited numerical simulation was carried out. The suggested sealing height is unlikely to be suitable for all types of masonry construction since the effect of different edge support conditions and vertical imposed loads due to multiple storey construction were not considered.

Building codes do not offer any specific guidance to allow the calculation of a safe maximum sealing height, yet government advice often suggests not to exceed 0.9 m [13–15]. Interim guidance given by the UK government suggested that structural damage would likely occur if a depth of water over 1 m was applied to the walls, but without justification [16], although examination of external walls was advised for expected flood depths of 0.6–0.9 m. Later guidance in the UK [17] reduced the safe working level to 0.6 m and direct reference was given to the work conducted by Pace [12], however the guidance seems contradictory to the maximum sealing height found in Pace’s study and is presented in the absence of published partial material and load factors.

Analytical work on the load capacity of masonry subject to non-uniform hydraulic lateral loads is limited. Masonry wall panels subject to uniform lateral loads have generally been modelled with acceptable correlation to the experimental failure patterns and failure loads using the yield line theory [18, 19]. Experimental studies include those conducted previously by the authors [19, 20] who used a geotechnical centrifuge to correctly model the masonry self-weight when working with uniformly laterally loaded small-scale masonry units. Theoretical failure loads, calculated via the established yield line analysis method, compared reasonably well to the experimental values. However, no such comparison between experimental and analytical models exists for the case of non-uniform lateral loading. Kelman and Spence [21] presented a yield line method to determine the flood load capacity of masonry wall panels. The yield line analysis was completed for a number of different sized wall panels, and solutions were determined numerically. It was concluded that structural failure would occur at water levels of between 1.0 and 1.5 m when no hydrodynamic effects were considered. Incorporating velocity into the calculations reduced the water level at failure to below 0.5 m. Standard guidance on design loads for masonry subjected to flooding is limited [13], simply referring to calculation methods for arching or two-way spanning wall panels as given for uniform wind loads. Whilst it has been suggested previously that the non-uniform loading may be replaced by a uniform load of equal magnitude, as directed in BS EN 1996-1-1:2005 [13], no such experimental tests appear to have been completed to verify this [22].

The lack of rigorous experimental data and consideration of realistic numerical models make it evident that there is a need to establish the safe water height capable of being restrained by typical masonry structures, and therefore the height to which waterproofing products should be applied when protecting against floodwaters.

This paper presents experimental data on the effect of out-of-plane hydrostatic loading on single skin masonry walls, typical of domestic or commercial premises. The experimental arrangement is based on that employed previously by the authors in the consideration of uniform lateral load on masonry panels [20]. In the current study two masonry panels each of autoclaved aerated Concrete (AAC) block, brick and brick-block units were constructed at small scale (1/6th) and hydraulic out-of-plane loading was applied incrementally to the masonry panels until failure occurred. The influence of mortar strength and panel support conditions, under constant axial load were examined. The study was completed using a geotechnical centrifuge to correctly model the effect of self-weight. 3D digital image correlation (DIC) was employed to monitor the wall panel during the tests and allowed in and out-of-plane deflections to be obtained. This paper also reports results from an analytical study in which a basic, but tractable, yield line approach was used to calculate water levels at failure for the wall panels considered in the experimental study. A comparison is then made between these water levels, the experimental water levels and those calculated according to BS EN 1996-1-1:2005 (EC6) [23], followed by further consideration of the safe maximum sealing height of waterproofing products to domestic dwellings.

## Experimental design

### Materials

Small scale (1/6th) masonry modelling using a geotechnical centrifuge has been justified and employed successfully by a number of researchers [24–27] and it allows testing to be completed economically and safely. Material selection is primarily driven by the need to accurately represent prototype material properties at the model scale. Cement-lime-aggregate mortars of two strength classes (M2 and M4) according to the National Annex to BS EN 1996-1-1:2005 [23] were used in this study, representative of mortars used in standard building practice in the UK.

CEM II cement, complying with BS EN 197-1 [28] was used together with hydrated lime of designation CL90-S, conforming to BS EN 459-1 [29]. Congleton HST95 aggregate with maximum particle size of 0.3 mm was used in the model scale mortar. This ensured that proper bedding of the masonry units could be achieved with the 2 mm mortar joint height employed at model scale. The water to cement ratio was adjusted for each mix to provide a suitable workability. Since good correlation between the compressive strengths of 25 and 70.6 mm mortar cubes has previously been reported [30], 25 mm mortar cubes were cast and tested using a 20 kN capacity test machine at a loading rate of 0.033 mm/s. Baggeridge Mellowed Red solid stock clay bricks from the same batch were used to form the brick and brick-block units. Standard grade Celcon AAC blocks conforming to BS EN 771-4 [31] were used to form the AAC block units.

### Masonry unit manufacture

Model scale brick units (35.8 mm long × 10.8 mm high × 17.1 mm deep) were cut from prototype brick units using a method developed by Hughes et al. [25]. Block units (73.3 long × 35.8 mm high × 16.7 mm deep) were cut from brick and AAC prototype units following the procedure outlined by Herbert et al. [20]. The cutting process ensured that the model and prototype units had consistent properties [25].

### Wall panel assembly procedure

**Fig. 1:**
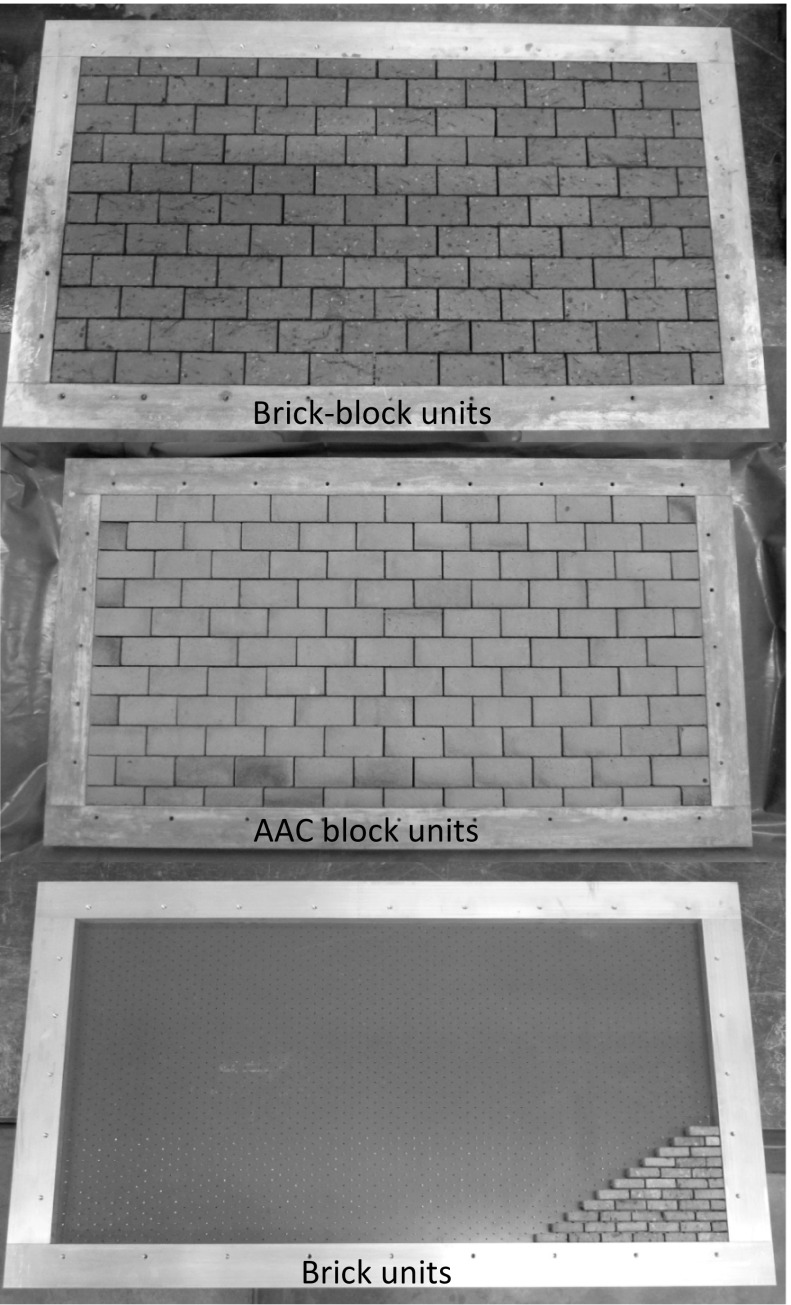
Construction of wall panels in bespoke jig for Brick-Block units; AAC Block units and Brick units

Wall panels of width 792 mm and height 408 mm (4.755 m × 2.452 m at prototype scale) were assembled using a bespoke jig, full details of which have been published by Herbert et al. [20]. The jig allowed the assembly of brick, brick-block and AAC block wall panels. The units were dry-assembled in the jig via placement between a series of locating pins (Fig. 1). Once full, the jig was placed in a water tank for 20 min, in order to pre-soak the units prior to mortar placement. The mortar was placed between the joints and compacted using light vibration, a process which was repeated until the mortar joints were full. A top plate was added to the jig and the whole jig arrangement was turned though 180° and the base plate of the jig removed. Voids left by the locating pins on the base plate were filled with mortar. The wall panel was covered with plastic film and left to cure for 28 days, although it was removed from the jig at 2 days. Two wall panels were constructed for each type of unit and mortar class as presented in Table 1.

**Table 1:** Schedule of wall panel tests

Specimen reference	Masonry type	Mortar type	Axial loading
H1	AAC block	M2^a^	1 storey
H2	AAC block	M2	1 storey
H3	Brick block	M2	1 storey
H4	Brick block	M2	1 storey
H5	Brick	M2	1 storey
H6	Brick	M2	1 storey
H7	Brick	M4^b^	1 storey
H8	Brick	M4	1 storey

^a^M2 Mortar with mix ratio by mass of 1:1:10 (Cement:Lime:Aggregate) with a 2.7 water cement ratio

^b^M4 Mortar with mix ratio by mass of 1:½:6½ (Cement:Lime:Aggregate) with a 1.8 water cement ratio

### Centrifuge testing details

**Fig. 2:**
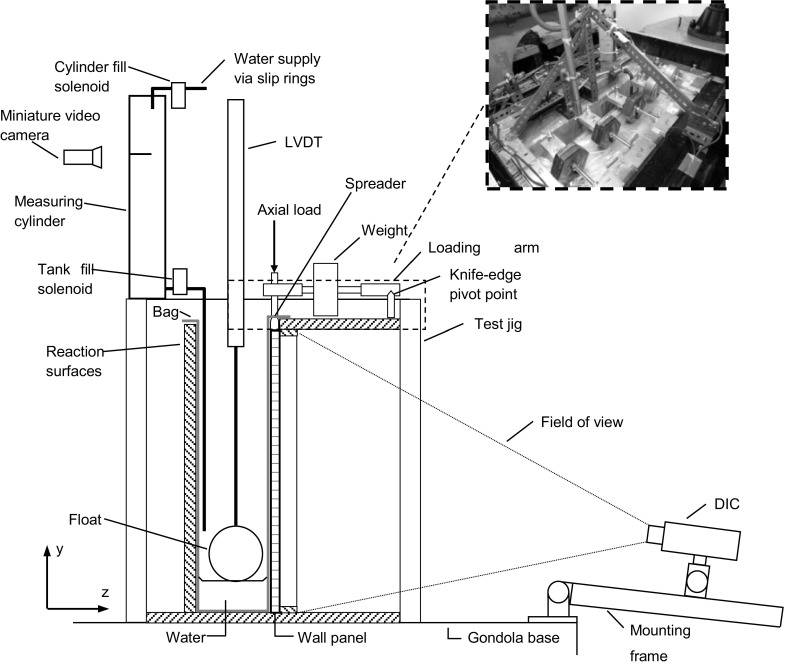
Non-uniform lateral loading test arrangement

Testing was completed using a centrifuge to ensure that a correctly scaled non-uniform load was applied to the wall panel specimens, as previously detailed by Herbert et al. [20]. A testing jig was designed to hold the specimen securely to the centrifuge gondola and provide a means of applying lateral hydraulic loading and axial load (Fig. 2). A loose fitting thin polythene bag was used to contain the water during test. Whilst the edges of the wall panels were simply supported it would be difficult to model the exact support conditions at the top of the wall panel at reduced scale, so the floor structure was assumed to provide a simple support to the wall panel in conjunction with a vertical axial load representative of one storey of loading (1.86–4.51 kN/m depending on unit type and density).

Water flow into the apparatus was remotely controlled via two solenoid valves. Flow was firstly directed to a measuring cylinder to enable precise control of the water level. A miniature video camera was used to monitor the water level in the measuring cylinder during the filling process. A second valve was used to release the water from the measuring cylinder into the water bag. This process ensured that the level increment at each fill was consistent. Measurement of the water level behind the specimen was made using a LVDT with a float attached. At the rear of the test jig a window was positioned upon which water level graduations were marked. A miniature video camera was positioned in front of the window to enable additional monitoring of the water level during the test.

In- and out-of-plane deflections of the wall panel were measured using a 3D DIC system. The set up of the system and details of calibration and operation are provided in full by Herbert et al. [19, 20]. A speckle pattern, required for DIC, was created on the wall panel after 21 days of curing, using a coat of matt white paint and fine tipped black marker pens. The DIC system was set to capture data every 10 s. The hydraulic loading was applied to the wall panel in 10 mm level increments and the out-of-plane deflection of the wall was allowed to stabilise before a further increment was applied. This process was continued until failure of the wall panel occurred, at which point the centrifuge was stopped and the wall panel was recovered from the test jig. Upon recovery, the crack pattern was recorded from the wall panel. Vic 3D was used to analyse images captured after each level increment to obtain displacements and contour plots of the wall panel during the test. The data from the water level sensor was combined with the displacements from Vic 3D to give the load–deflection response of the wall panel. Unlike discrete LVDT measurements, the DIC system also allowed the position and development of cracks to be identified during the loading process.

## Experimental results and discussion

### Materials

The average compressive strengths of the M2 and M4 mortar cubes for all specimens are presented in Table 2. Consistent compressive strength results, irrespective of mortar strength, were obtained which were above those suggested by the prescribed mixing ratio.

**Table 2:** Average compressive strength results of mortar cubes

Specimen reference (mortar type)	Compressive strength (N/mm^2^)	Coefficient of variation (%)
H1 (M2)	2.61	11.28
H2 (M2)	3.06	3.82
H3 (M2)	2.81	12.09
H4 (M2)	3.24	3.16
H5 (M2)	3.19	3.85
H6 (M2)	2.80	0.69
H7 (M4)	7.87	3.71
H8 (M4)	7.89	6.04

### Block specimens

**Fig. 3:**
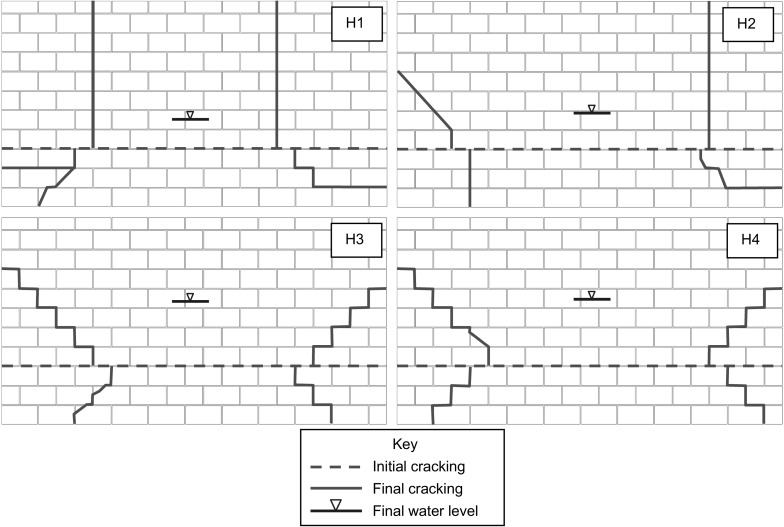
Crack patterns for all block wall panels

The failure modes observed for the AAC block (H1 and H2) and brick block (H3 and H4) wall panels, constructed with M2 compressive strength mortar, are shown in Fig. 3. Initial horizontal cracking was observed to occur in the lower section of the wall panels at a height approximately equal to the midpoint of the final water level. Cracks tended to form through the units and mortar joints for the AAC block panels (H1 and H2), whilst were generally restricted to the mortar joints for the brick block specimens (H3 and H4) reflecting the relative strengths of the blocks and mortar.

**Fig. 4:**
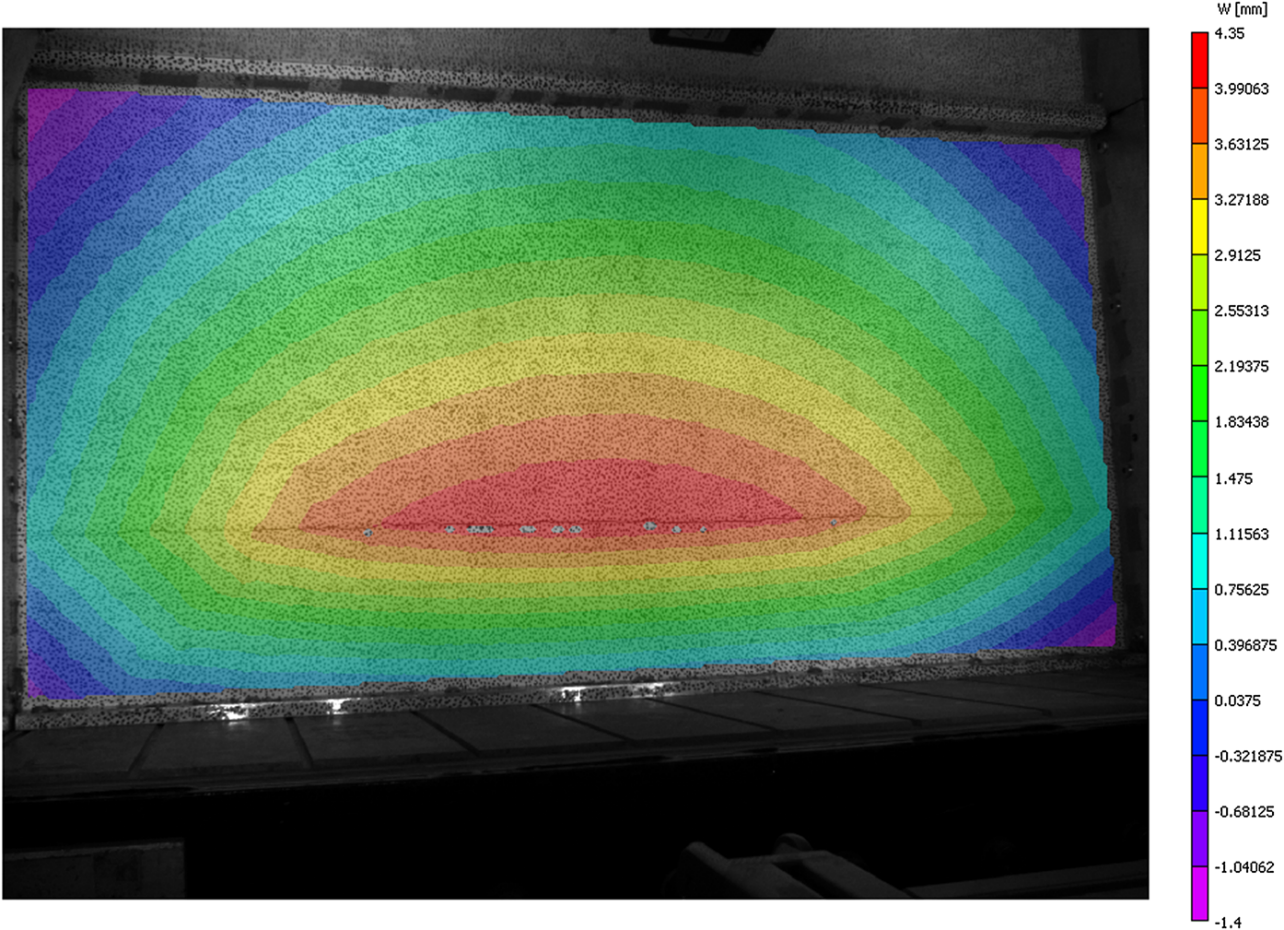
Contour plot of *z*-deflections for brick-block specimen (H4) immediately prior to failure

Failure of the wall panels was rapid, thus making it difficult to capture the process between formation of the final crack pattern and actual failure. This is in contrast to the progressive behaviour of wall panels subjected to uniform loading as reported by Herbert et al. [20]. Negative deflection at the corners of the panels was observed immediately prior to failure when the final crack pattern was evident (Fig. 4). In the bottom section of the panel, this was likely due to the cracks not forming into the corners and a pivoting type action occurring about the supports. Similar behaviour only occurred in the upper sections of specimens H1 and H2 where the cracks formed in a direction towards the horizontal upper support. In specimens H2 (left edge), H3 and H4 the diagonal cracks tended towards the side supports in the upper section, such that negative deflection could only occur by curvature of this part of the panel and not by a pivoting effect.

**Fig. 5:**
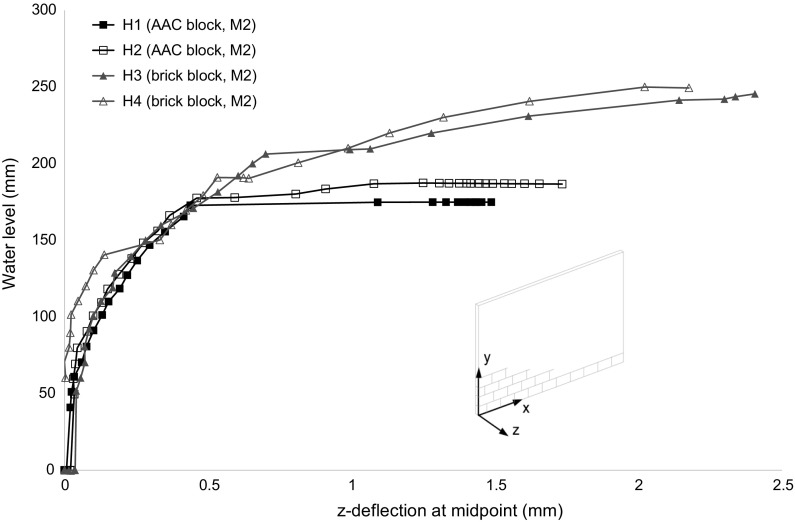
Load versus *z*-deflection at centre of wall panel for hydraulically loaded block specimens (experiments H1–H4)

Out-of-plane (*z*) deflections at the centre of the hydraulically loaded block wall panels (Fig. 5) showed that pre-initial cracking load deflection behaviour comprised an initial linear stage, up until a water level of approximately 80 mm, followed by a curved response, where the stiffness gradually reduced as the water level increased. This response was generally similar for all specimens, with the exception of H4, where the specimen moved back from the supports during the centrifuge start up procedure. It was not possible to apply any pre loading to the wall and it was difficult to maintain full contact prior to loading without affecting the actual support conditions.

Peak deflections prior to failure were on average 40% of those found in uniform loading tests on identical wall panel arrangements [19]. It is likely that the position of the initial crack influenced the peak deflection, along with the speed of the failure process. The section of the wall panel below the crack would be subject to higher in-plane rotations about the hinges at identical deflections as its height reduced. This would result in the lower section of the wall panel becoming unstable at lower levels of deflection, resulting in a more rapid progression to failure.

Very limited information was given for the only previously completed tests for hydraulic loading on concrete block conducted at prototype scale, with regards to the unit dimensions, type of mortar or block used [12]. The results may also be inconclusive, since the tests were stopped before failure due to severe water leakage through the walls. The load–deflection response shown was however similar to that of specimens H1 and H2 with no residual capacity after an initially curved behaviour. The peak level of 1.10 m was of a similar magnitude to the scaled mean of specimens H1 and H2 at 1.09 m. Pace [12] also suggested that 0.61 m was a safe water level for blockwork walls, but without any reasoning or justification. The results shown here illustrate that the block strength affected the peak water level and one safe level for all would clearly not be appropriate.

### Brick specimens

**Fig. 6:**
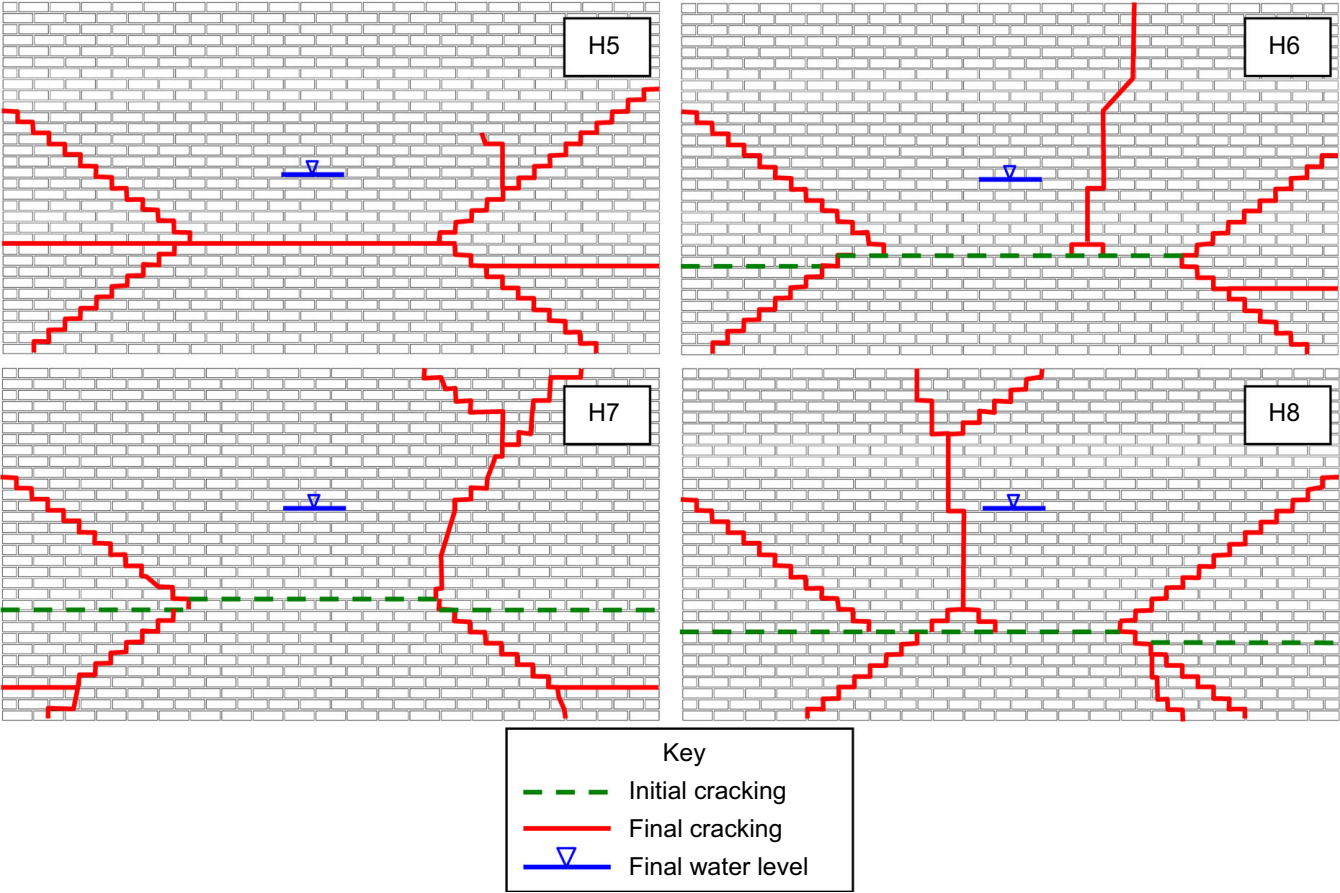
Crack patterns for all brick wall panels

The failure modes for brick wall panels constructed with M2 (H5 and H6) and M4 mortar (H7 and H8), are shown in Fig. 6. The test conditions (edge supports and imposed vertical axial load) for the specimens were otherwise identical. Cracking was found to initiate horizontally across the wall panels in the lower section and was generally near to the mid-point of the peak water level. The position of the initial crack was also found to be at a height of approximately double the height to the centroid of the hydraulic loading profile at the time of cracking. For specimen H5 no cracking was apparent in the specimen prior to failure occurring and horizontal and diagonal cracking occurred simultaneously. At failure there was typically a combination of diagonal cracking through the mortar joints only and cracking through the units and mortar joints. The crack patterns were similar for M2 and M4 mortar strengths, although it was apparent that some additional cracking occurred in the panels with M4 mortar at failure. Some similarities were observed with the failure modes of the brick block specimens discussed previously, however in the brick block specimens cracking was generally restricted to the mortar joints only.

As with the block specimens, failure was rapid. Negative deflection at the corners of the panels was generally observed both immediately prior to and after failure.

**Fig. 7:**
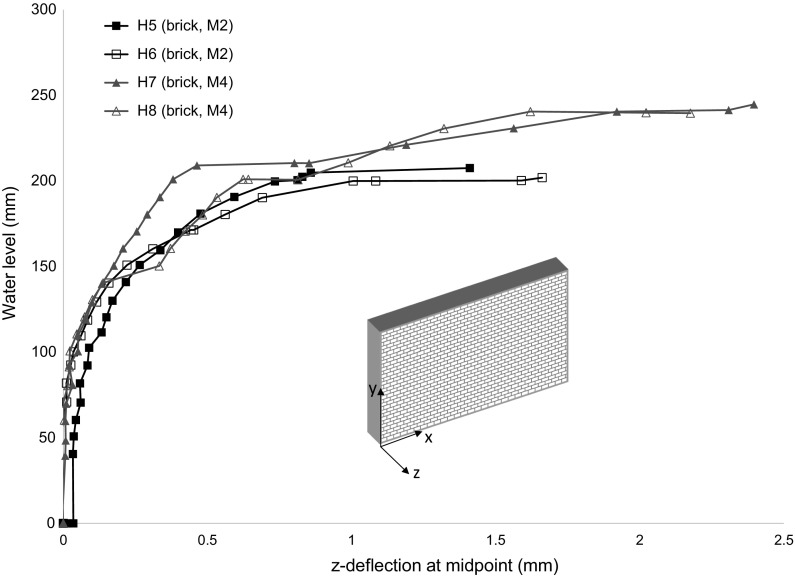
Load versus *z*-deflection at centre of wall panel for hydraulically loaded brick specimens (H5–H8)

The load versus *z*-deflection responses for the brick wall panels constructed with M2 and M4 compressive strength mortar (Fig. 7) were initially approximately linear and of similar stiffness for all specimens regardless of mortar strength up until a water level of 100 mm. Following this, the stiffness of the specimens tended to decrease in a non-linear manner until initial cracking was evident. For the wall panels constructed with M2 mortar (H5 and H6) initial cracking and failure was at approximately constant load and no residual strength was apparent. The response for specimens H5 and H6 was very similar despite the slight differences observed in the failure modes.

The pre-cracking response of the wall panels constructed with M4 mortar (H7 and H8) was similar until a water level of 140 mm where a step change occurred (H8) likely due to bedding in at the upper support. The response that followed was however of comparable stiffness and both specimens showed residual strength capacity following initial cracking. It was found that as the mortar strength was increased there was an average 19% increase in the water level at failure, although the water level at initial cracking was similar. The ability of the higher mortar strength specimens to sustain further loading was likely due to the increased flexural strength in the direction perpendicular to the bed joints (*f*_*xy*_).

### Consideration of variability

The repeat specimens consistently failed at very similar water levels (Table 3), with consistently low coefficients of variation. In the only previous experimental study of blockwork walls subject to hydraulic loading [12] no repeats were conducted so it is difficult to establish the typical variation expected between specimens. No information was available in the literature to compare brick specimens. Although comparison with uniform loading tests should be treated with caution due to the different loading profiles, similarly small variation was found by Herbert et al. [20] on small-scale brick specimens. However, de Vekey et al. [32] found that in tests on AAC blockwork variability was much higher (repeat specimens within 14% on average) whilst with brick specimens an average difference between experimental failure loads reported by West et al. [33] was 39%.

**Table 3:** Summary of failure conditions (at 1/6th scale)

Specimen reference	Water level (mm)		Final failure	
	Initial cracking	Final failure	Mean water level (mm)	Coefficient of variation (%)
H1 (AAC block, M2)	175	175	181	4.69
H2 (AAC block, M2)	180	187		
H3 (brick block, M2)	209	245	248	1.43
H4 (brick block, M2)	191	250		
H5 (brick, M2)	208	208	205	2.07
H6 (brick, M2)	202	202		
H7 (brick, M4)	210	247	244	2.03
H8 (brick, M4)	201	240		

## Application of yield line analysis to masonry subject to hydraulic loading

Masonry wall panels subject to uniform lateral loads have generally been modelled with yield line theory utilising flexural strength models that have provided acceptable correlation between the experimental failure patterns and failure loads [18, 33–38]. It is appreciated that aside from standard yield line analysis, there are a number of approaches available to simulate the out-of-plane response of masonry panels. These involve consideration of macro- and micro- mechanical mechanisms [39, 40] and the use of homogenization techniques [41–47], all of which have provided robust alternatives to describe the behaviour of masonry panels subject to out-of-plane loads. Nevertheless, the role of the analysis in the current study was to support the interpretation of the experimental results and to assess the ability of a standard yield method to predict failure water levels [21]. This solution was implemented in a Microsoft Excel worksheet such that it could later be adopted as an analysis tool accessible to a wide range of industry practitioners.

**Fig. 8:**
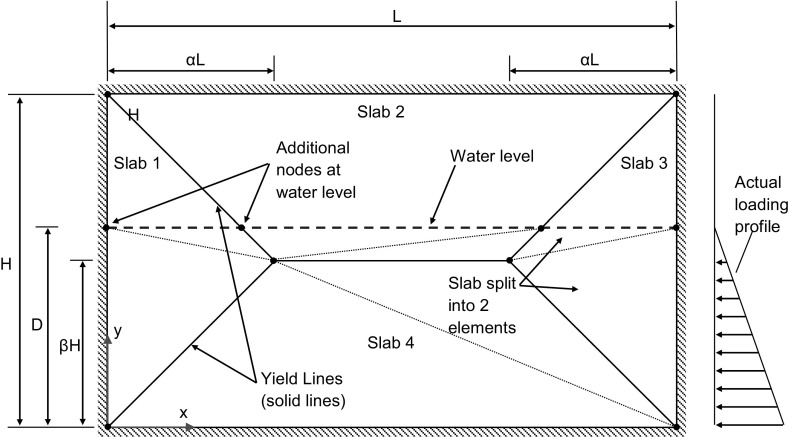
Assumed yield line pattern and loading profile for wall panel with slabs split into additional elements

To apply yield line theory to a masonry wall panel a suitable crack pattern must first be postulated for the problem. For the case of a wall panel simply supported on all edges, an envelope type failure pattern is assumed, as shown by Fig. 8. The standard yield line pattern adopted is informed by experimental observations from our own tests, as well as those from Kelman and Spence [21] and Sinha [34]. It is acknowledged that a general procedure for identifying optimum yield patterns requires a more complex approach such as the Sequential Linear Programming algorithm adopted by Milani [48].

### Moment capacities and the computation of internal and external components of virtual work

**Fig. 9:**
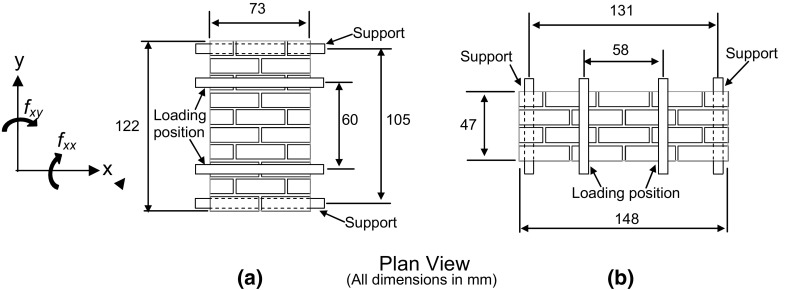
Model scale flexural testing arrangement for brick units for a plane of failure **a** parallel to bed joints (*f*_*xx*_) and **b** perpendicular to the bed joints (*f*_*xy*_) (all dimensions in mm)

The wall is assumed to have a moment capacity in the two orthogonal directions associated with the Cartesian axis coordinates (*x* and *y*). The moment resistances are presented as the effective flexural strengths multiplied by the section modulus (Eqs. 1 and 2). However, it should be noted that this does not imply that the system was elastic at the time of failure, but these effective strengths are merely used as a simple comparative way of expressing the ultimate flexural strength. The moment resistance about the *x*-axis (*M*_Rdy_) is enhanced to allow for vertical load effects and is calculated following the procedure introduced by Kelman and Spence [21], as adopted in the yield line analysis of wall panels subject to uniform lateral loading by the current authors [19]. By contrast, the moment resistance about the *y*-axis (*M*_Rdx_) is based on the flexural strength of the masonry alone. The basic flexural strengths of the masonry in the two orthogonal directions were obtained from a series of experimental wallette tests, as initially proposed by Sinha [34], and described and presented by Herbert et al. [19] and Herbert [50]. An example arrangement of the wallette tests for brick units is given in Fig. 9.

1 $$M_{\text{Rdy}} = (f_{xx} + \sigma_{yy} )Z$$

2 $$M_{\text{Rdx}} = f_{xy} Z$$

in which *f*_*xx*_ is the flexural strength of the masonry about the *x* axis, *σ*_*yy*_ is the average vertical stress at the yield line position, *f*_*xy*_ is the flexural strength of the masonry about the *y* axis and *Z* the section modulus. *f*_*xx*_*, f*_*xy*_ and *σ*_*yy*_ are taken as positive in compression in Eqs. (1) and (2).

The internal work associated with each yield line region was computed using the Jones–Wood method [49], with the orthogonal capacities in the *x* or *y* directions being considered in turn. The internal work associated with each region and direction is then the product of the appropriate moment resistance per unit width (*M*_Rdx_ or *M*_Rdy_), the projected length of the yield line -perpendicular to the respective direction- and the rotation of the yield line about the axis under consideration.

To calculate the external work done on the slab, the yield line regions were subdivided (if necessary) into a series of triangular elements, or sub-regions. The hydrostatic pressure (*P*_ihs_) at each node of a triangular element was determined from the depth of the water (*D*) according to Eq. (3);

3 $$P_{\text{ihs}} = \rho_{w} g(D - y_{i} )$$

where $\rho_{w}$ is the density of water, *g* is the gravitational constant, and *y*_*i*_ is the ordinate of the node under consideration, with reference to the base of the wall panel, as shown in Fig. 8.

The external work on each element was then computed from the integral of the product of the pressure and the virtual displacement field. The components of external work on each element were then summed to give the total for each region and these, in turn, were summed to provide the total external work.

The resulting expressions for the total external work and internal work were then equated. Using the resulting expression, the optimum yield line pattern and failure depth were determined by simultaneously optimising the parameters that determine the position of the yield lines (e.g. *α* and *β* in Fig. 8) and the water depth (*D*).

### Comparison of experimental and analytical results

**Fig. 10:**
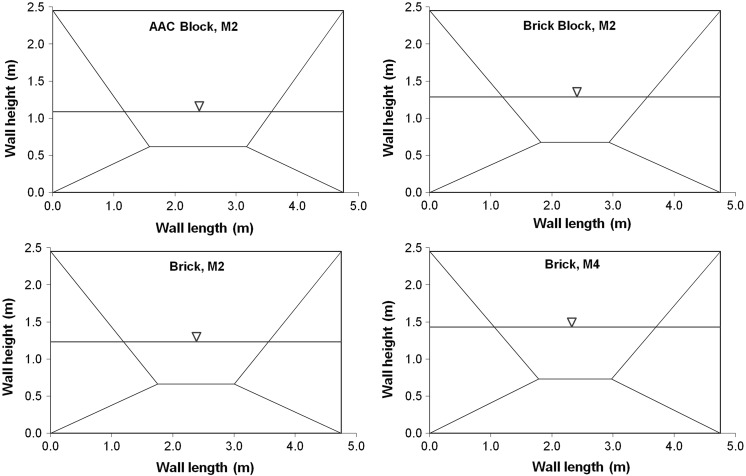
Yield line patterns and water level at failure using flexural strengths determined from wallette tests

Using the above procedure, the analytical water levels at failure were calculated for the wall panels considered in the experimental study using the parameters detailed in Table 4. The analytical model was completed at prototype scale and the experimental failure levels presented in Table 5 were scaled by the scaling factor of 6. Two sets of analyses were undertaken to determine the water level at failure. Analysis (1) used the enhanced flexural strength (*f*_*xx*_ + *σ*_*yy*_) and *f*_*xy*_ from the experimental wallette tests and analysis (2) used EC6 adjusted flexural strengths, as discussed by Herbert et al. [19] and Herbert [50]. The parameters used in both analyses are summarised in Table 4. The water levels at failure for the wall panels H1 to H8 are given in Table 5 and their associated yield line patterns are presented in Fig. 10.

**Table 4:** Parameters used in the analysis

Masonry/mortar type	Masonry unit thickness (m)	Masonry unit density (kg/m^3^)	Vertical load at top of wall (kN/m)	Experimental flexural strength (N/mm^2^)		Adjusted EC6 flexural strength (N/mm^2^)	
				*f*_*xx*_	*f*_*xy*_	*f*_*xx*_	*f*_*xy*_
AAC block, M2	0.100	772	1.86	0.41	0.67	0.30	0.60
Brick block, M2	0.100	1828	4.40	0.50	1.28	0.53	1.50
Brick, M2	0.103	1828	4.51	0.44	1.02	0.53	1.50
Brick, M4	0.103	1828	4.51	0.66	1.45	0.60	1.65

**Table 5:** Comparison of experimental and analytical water levels at failure for single leaf panels

Specimen reference	Masonry/mortar type	Experimental water level at failure at prototype scale (m)		Analytical water level at failure based on flexural strength (m)	
		Individual	Average	Experimental	Adjusted EC6
H1	AAC block/M2	1.05	1.09	1.09	0.99
H2	AAC block/M2	1.12			
H3	Brick block/M2	1.47	1.46	1.29	1.34
H4	Brick block/M2	1.50			
H5	Brick/M2	1.25	1.23	1.23	1.37
H6	Brick/M2	1.21			
H7	Brick/M4	1.48	1.46	1.43	1.43
H8	Brick/M4	1.44

It was observed from (Table 5) that the analytical water levels at failure that were determined using the average flexural strengths from the wallette tests compared well to the experimental values, with the exception of the brick block specimens H3 and H4 that was underestimated by 14% in the analysis. The analytical water levels at failure determined from the EC6 adjusted flexural strengths, similarly, compared well to the experimental failure loads. The analysis again underestimated the failure level of the brick block specimens (H3 and H4), but by a lesser amount than previously, due to the EC6 adjusted flexural strengths exceeding those found in the wallette tests. The variation in the results for specimens H5 and H6 was similarly related to differences between the experimental wallette and EC6 adjusted flexural strengths.

The position of the yield lines in the analysis was generally considered to be an acceptable representation of the experimental failure modes, considering the constraints utilised in the analysis. The main differences in the failure modes that were observed in the optimised and experimental cases was that corner levers were observed to form in the specimens due to the position of the yield lines. A further refinement of the analysis to account for the formation of corner levels is the subject of an ongoing study.

In order that they can be compared directly to the experimental failure loads, the analytical results presented thus far have not included any partial material or load factors. The limit state design utilised in the analysis however requires the application of these factors to establish the safe maximum working water level that can be retained from the failure level determined for the wall panel. It is therefore important to establish the safe working levels for the experimentally tested wall panels to allow comparison to values given in the literature to evaluate the appropriateness of current guidance.

**Fig. 11:**
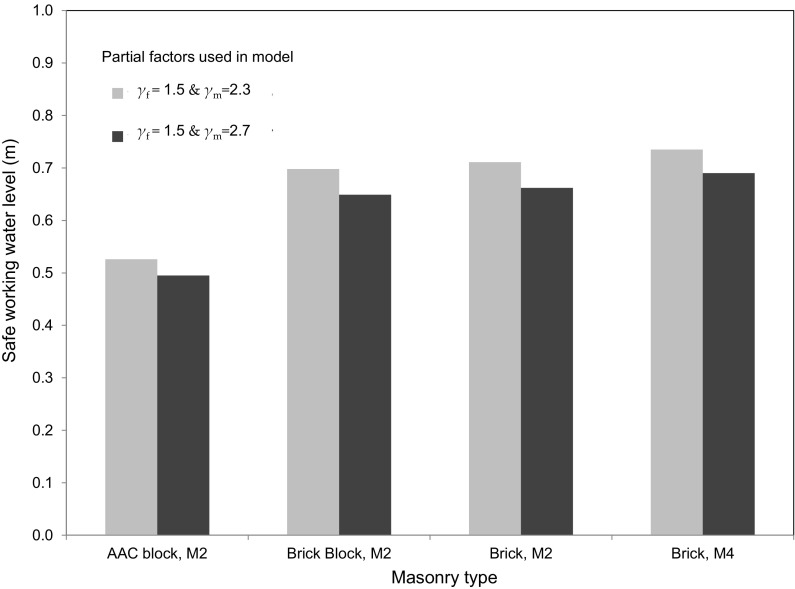
Safe working water levels for experimentally tested panels

The partial factor for the load (*γ*_f_) is prescribed as 1.5 in BS EN 1990: 2002 [51], whilst the partial factor for the materials (*γ*_m_) can take a value of either 2.3 or 2.7 depending on the quality of the construction of the masonry according to the National Annex to Eurocode 6 [23]. The safe working water levels were computed using the pre-adjusted EC6 [23] characteristic flexural strengths in the analysis. The characteristic flexural strengths were used rather than the experimental values to ensure the approach was in line with the calculation method given in EC6 for uniform loading conditions. The safe working water levels calculated for the hydraulically loaded specimens are shown in Fig. 11.

All specimen configurations failed to attain a safe working level of 0.9 m, regardless of the level of quality control imposed during construction. All specimens attained a safe working level of 0.6 m or more, with the exception of the AAC block specimen (H1 & H2), for which safe working levels of 0.53 and 0.49 m were computed for partial material factors of 2.3 and 2.7 respectively. The results clearly illustrated that for the particular conditions imposed in the experimental tests that a safe level of 0.9 m quoted in the literature [12, 16, 52] would generally be inappropriate, whilst the safe level of 0.6 m was not suitable for every case. It is recommended that providing such fixed values in the literature should be avoided and a limit state design using the analysis developed herein would offer a much more suitable approach than specifying a universal safe working level that has no regard for the particular conditions for the wall in question.

## Conclusions

Hydraulic loading on 1/6th scale wall panels was successfully achieved through the use of a geotechnical centrifuge. The failure modes of the wall panels were observed to be comparable between repeat specimens, although were not generally identical. Such differences were expected due to the inherent variation in the masonry units and the bond between the mortar and unit. Failure of the specimens was generally found to occur rapidly and often without warning. The behaviour differed to that found in uniform loading tests, where failure was more progressive. Initial cracking and failure were typically coincident for the AAC block and brick specimens constructed with M2 mortar. Residual strength was observed in the brick block, M4 mortar strength brick.

The peak water levels were very similar for the repeat specimens for all combinations of materials and mortar proving the repeatability of the manufacturing and testing procedure. The variability in the results was at the lower end of those reported in the literature for similar materials and mortars, although these were subject to uniform lateral load.

Cracking was generally limited to the mortar joints in the brick block specimens, but occurred both in the units and mortar joints in the AAC block panels. The initial response and stiffness was similar for all block specimens, but post cracking residual strength was observed in the brick block specimens. The peak water level was significantly higher for the brick block wall panels compared to those constructed with AAC block.

The results from the non-uniform hydraulic loading tests were used to verify the yield line analysis approach adopted and generally a good correlation was given in terms of water level at failure when the average flexural strengths were utilised. The water levels at failure calculated using the EC6 adjusted (to average) flexural strengths correlated well to the experimental test results, however the strength of the brick M2 compressive strength mortar wall panels (H5 and H6) were overestimated. It was suggested that where possible the appropriate flexural strengths should be utilised in analysis, although the EC6 characteristic values may be used that allow for greater degree of variability in the flexural strengths.

The analysis did not always predict the correct failure mode, particularly where corner levers were found to form. Nevertheless, the use of the analysis developed in the current study is acceptable for determining the collapse loads and would likely result in slightly conservative loads or water levels at failure. When partial material and load factors were incorporated into the analysis none of the wall panels tested could meet the requirements of the upper limit (0.9 m) specified in the published guidance, whilst the AAC block walls (H1 and H2) failed to meet the lower value given (0.6 m). This supports the need for a suitable approach for the calculation of water levels at failure rather than the use of fixed values given in published literature.
